# 

**Published:** 1892-05-28

**Authors:** 


					n?o?rJce twopence.;;
W Vol. XII.?May 28th, 1892.
ttaS?Tarthe General Post Office as a Newspaper for Mz*
^?"Mion in the United Kingdom and Abroad.! ,
IWITHCEXTRAS.NURSINGV SUPPLEMENT.
Per. Annum, 8s. 6d? post-free. Jfl^
Monthly IParts,56d.; post Eree,^8d.
Ditto per Annum, 8a., post free.
Vol. XII.] Saturday,
May 28th, 1892.
[Twopence.

				

